# Zinc as a Dual-Condition Inhibitor of HIF-1α/VEGF-α–Mediated Angiogenesis in Clear Cell Renal Carcinoma

**DOI:** 10.15586/jkc.v12i4.429

**Published:** 2025-10-28

**Authors:** Guilherme Oliveira Carlos, Beatriz Miquilino Neto, Luiz Felipe S. Teixeira, Amanda Sena de Sousa, Monica Beatriz Mathor, Maria Helena Bellini Marumo

**Affiliations:** 1 Biotechnology Center, Nuclear and Energy Research Institute (IPEN/CNEN), São Paulo, Brazil;; 2 Radiation Technology Center, Nuclear and Energy Research Institute (IPEN/CNEN), São Paulo, Brazil

**Keywords:** Angiogenesis, Clear cell renal cell carcinoma, HIF-1α, Hypoxia, Zinc

## Abstract

Clear cell renal cell carcinoma (ccRCC) is marked by aberrant hypoxia-driven signaling and enhanced angiogenesis mediated by hypoxia-inducible factor 1-alpha (HIF-1α) and vascular endothelial growth factor alpha (VEGF-α). Zinc (Zn), an essential trace element with emerging anticancer potential, was evaluated for its ability to modulate angiogenesis in von Hippel–Lindau (VHL)-deficient 786-0 cells under normoxic and hypoxic conditions. Using quantitative real-time polymerase chain reaction (qRT-PCR), Western blotting, enzyme-linked immunosorbent assay (ELISA), and immunofluorescence, we observed that Zn treatment reduced HIF-1α expression and VEGF-α secretion across both oxygenation states. Notably, Zn inhibited the hypoxia-induced nuclear accumulation of HIF-1α and attenuated paracrine endothelial activation, as shown by reduced human umbilical vein endothelial cell (HUVEC) viability in conditioned media assays. These effects likely involve transcriptional repression, enhanced proteasomal degradation of HIF-1α, and interference with VEGF-α–dependent signaling. Overall, our findings suggest that zinc may function as a multifunctional modulator of tumor angiogenesis and holds potential as an adjuvant in antiangiogenic strategies, particularly under hypoxic conditions.

## Introduction

Renal cell carcinoma (RCC) accounts for approximately 2.4% of all adult malignancies, totaling 434,840 new cases and 179,368 deaths worldwide in 2022 (1). Clear cell RCC (ccRCC) is the most common histological subtype, representing ~75% of cases, and is characterized by high metastatic potential and resistance to conventional therapies (2,3).

A hallmark of ccRCC is the frequent loss of function of the von Hippel–Lindau (VHL) tumor suppressor gene, which leads to hypoxia-inducible factor (HIF) stabilization and overexpression of vascular endothelial growth factor (VEGF) and thereby promoting angiogenesis (4–6). Hypoxia-driven HIF-1α accumulation and subsequent VEGF-α transcription are strongly correlated with the intense vascularization and aggressiveness observed in ccRCC (7,8).

Targeting the HIF/VEGF axis remains a central therapeutic strategy in ccRCC. However, the precise role of micronutrients such as Zn in this pathway remains poorly defined. Zinc is an essential trace element involved in enzymatic regulation, transcriptional control, and oxidative balance (9). Previous studies have suggested that zinc may destabilize HIF-1α and reduce VEGF-α expression, even under hypoxic or VHL-deficient conditions (10,11). In addition, zinc has been reported to induce autophagy, modulate immune responses, and exert dual effects on tumor progression depending on concentration and cellular context (12–15).

Given this background, the present study aimed to investigate the modulatory effects of zinc on HIF-1α and VEGF-α expression in 786-0 ccRCC cells under normoxic and hypoxic conditions.

## Materials and Methods

### Cell Lines and Culture Conditions

The human clear cell renal adenocarcinoma cell line 786-0, which carries a VHL gene mutation, was obtained from the American Type Culture Collection (ATCC^®^ CRL-1932; Manassas, VA, USA). Cells were cultured in Roswell Park Memorial Institute (RPMI)-1640 medium (Life Technologies, Carlsbad, CA, USA) supplemented with 10% fetal bovine serum (FBS), 100 U/mL penicillin, and 100 µg/mL streptomycin (all from Gibco, Waltham, MA, USA).

Human umbilical vein endothelial cells (HUVECs) were purchased from Gibco (Gibco^®^, Thermo Fisher Scientific, Waltham, MA, USA) and cultured in Opti-MEM^®^ reduced serum medium supplemented with 5% FBS, 100 U/mL penicillin, and 100 µg/mL streptomycin.

Cells were plated in 100 mm Petri dishes and maintained in a humidified chamber at 37°C with 5% CO_2_.

### ZnCl_2_ Supplementation

Zinc chloride (ZnCl_2_; Merck, Darmstadt, Germany) was prepared as a 100 mM stock solution in ultrapure water (Milli-Q^®^ system; Sigma-Aldrich, Burlington, MA, USA), sterilized using a 0.22 µm membrane filter, and stored at 4°C. For all assays, ZnCl_2_ was freshly diluted to a final concentration of 100 µM in RPMI 1640 complete medium immediately prior to use.

Cells were treated with ZnCl_2_ under both normoxic and hypoxic conditions for 24, 48, or 72 hours depending on the experiment (qRT-PCR, Western blotting, ELISA, immunofluorescence, or conditioned media for HUVEC assays). Control cells were maintained under the same conditions without ZnCl_2_. Medium was refreshed every 24 hours.

### Cell Viability and Cytotoxicity Assay: Dose–Response Curve

Cell viability was assessed using the MTS assay (CellTiter 96® AQueous One Solution Cell Proliferation Assay Kit; Promega^®^, Madison, WI, USA), which quantifies mitochondrial activity via reduction of the tetrazolium compound MTS [3-(4,5-dimethylthiazol-2-yl)-5-(3-carboxymethoxyphenyl)-2-(4-sulfophenyl)-2H-tetrazolium] into a red formazan product, with absorbance measured at 490 nm.

Cells were seeded in 96-well plates (5 × 10^3^ cells/well) in quintuplicates and incubated for 24 hours. ZnCl_2_ was then applied at final concentrations of 60, 80, 100, and 120 µM, followed by another 24-hour incubation. After treatment, medium was replaced with 100 µL of RPMI 1640 (supplemented with 10% FBS, 300 µg/mL streptomycin, and 100 U/mL penicillin) plus 20 µL of the MTS reagent. Plates were protected from light and incubated for 2 hours at 37°C. Absorbance was measured at 490 nm using a Multiskan EX microplate reader (Labsystems, Milford, MA, USA).

### Hypoxia Induction

Hypoxic conditions (<1% O_2_) were achieved by sealing cultured cells in a plastic container (20 × 14.9 × 4.7 cm) with three oxygen absorber sachets (6 g each; QuimicSul, Joinville, SC, Brazil) and heat-sealing. An additional sachet was placed in a second sealed bag within the same container to enhance O_2_ depletion. Incubation was conducted at 37°C with 5% CO_2_ for 8 hours. This approach was adapted from established hypoxia protocols using scavenger sachets.

This method reproducibly achieves oxygen levels of approximately 0.5–1% O_2_, as validated by previously established protocols using scavenger sachets (16).

### RNA Extraction and Quantitative Real-Time PCR (qRT-PCR)

Total RNA was extracted using an RNeasy Mini Kit (Qiagen, Valencia, CA, USA). cDNA synthesis was performed using the QuantiTect^®^ Reverse Transcription Kit (Qiagen, Hilden, Germany) with 2 µg RNA. Reactions were performed with Absolute SYBR Green qPCR Mix^®^ (Invitrogen) in 10 µL volume on an ABI Prism 7000 system (Applied Biosystems).

Thermal cycling: 95°C for 15 seconds, 60°C for 1 minute, 72°C for 1 minute (40 cycles). TFRC was used as the reference gene.

Primers:

- HIF-1α: Fwd 5’-TTTACCATGCCCCAGATTCAG-3’; Rev 5’-GGTGAACTTTGTCTAGTGCTTCCA-3’;
- VEGF-α: Fwd 5’-CGAGGGCCTGGAGTGTGT-3’; Rev 5’-CCGCATAATCTGCATGGTGAT-3’;
- TFRC: Fwd 5’-GGAGGACGCGCTAGTGTTCT-3’; Rev 5’-TGCTGATCTAGCTTGATCCATCA-3’.

Expression was calculated using the 2^-ΔΔCt^ method.

### Protein Extraction and Western Blotting

Proteins were extracted with CelLytic™ reagent (Sigma-Aldrich), and supernatants were collected after centrifugation at 20,000 × g for 15 minutes at 4°C. Protein concentration was determined via the bicinchoninic acid method. SDS-PAGE was performed with 40 µg protein/lane. Proteins were transferred to PVDF membranes (ThermoFisher) and blocked with 5% skim milk in TBS.

Primary antibodies (1:500) used: anti-GAPDH (sc-365062, mouse), anti-HIF-1α (ab8366, mouse), anti-VEGF-α (sc-7269, mouse). Secondary antibody used: goat anti-mouse IgG HRP-conjugated (a4416; 1:1000; Sigma). Detection: SuperSignal^®^ West Pico kit (Thermo Fisher). Images were acquired with the Uvitec Cambridge Alliance 4.7 system.

### Enzyme-linked Immunosorbent Assay (ELISA)

VEGF-α levels in culture supernatants were measured with the Human VEGF ELISA Kit (RAB0507, Merck). Samples were obtained from 786-0 cells treated with ZnCl_2_ for 24–72 hours. Supernatants were centrifuged (1000 × g, 5 minutes, 4°C) and stored at −80°C. Absorbance was read at 450 nm. Concentrations were calculated using a standard curve.

### Immunofluorescence

Cells were fixed with 4% paraformaldehyde (10 minutes), blocked with 1% BSA, and incubated with primary antibodies: anti-HIF-1α, anti-VEGF-α (Santa Cruz), and anti-α-tubulin (Cell Signaling) at 1:100. Secondary antibodies: Alexa Fluor 488 (for α-tubulin) and Alexa Fluor 568/594 (for HIF-1α and VEGF-α), all at 1:200.

DAPI mounting medium (ProLong Gold with DAPI; Invitrogen) was used. Images were captured using the TissueFAXS i PLUS system and analyzed in ImageJ.

### HUVEC Medium Conditioning

HUVECs (ATCC^®^ CRL-1730) were cultured in Opti-MEM with 5% FBS and progressively adapted to RPMI 1640. Once adapted, cells were maintained at 37°C under normoxia with 5% CO_2_.

### HUVEC Viability Assay with Conditioned Medium

HUVECs were seeded in 96-well plates (5 × 10^3^ cells/well). Conditioned media from ZnCl_2_-treated 786-0 cells were applied for 24 hours. Cell viability was assessed with CellTiter 96^®^ AQueous One Solution assay (Promega) and absorbance at 490 nm.

### Statistical Analysis

Data were analyzed using GraphPad Prism 6.0 (GraphPad Software Inc., La Jolla, CA, USA). Results are presented as mean ± SEM. Comparisons were performed using Student’s *t*-test or one-way ANOVA with Bonferroni’s post-hoc test. All experiments were performed with n = 3 biological replicates. P-values are reported in figure legends, with significance thresholds as follows: *p < 0.05, **p < 0.01, ***p < 0.001, and ****p < 0.0001.

## Results

### Zinc Modulates 786-0 Cell Viability

In this study, we performed in vitro experiments using cell lines derived from human renal tumor tissue (786-0) and normal endothelial cells (HUVEC). In all experimental conditions, cell viability remained consistently above 95% in both cell lines, confirming the absence of cytotoxic effects from the treatments and ensuring experimental reliability.

**Figure 1:**
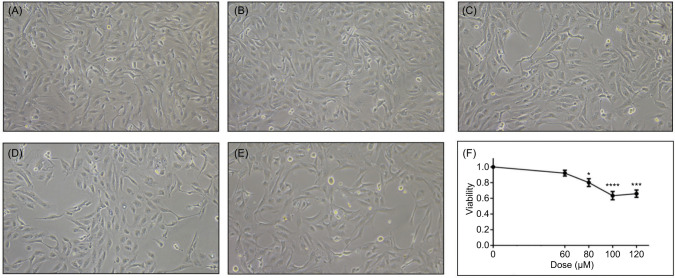
Zinc treatment modulates viability of 786-0 cells. (A–E) Representative phase-contrast microscopy images of 786-0 renal carcinoma cells cultured under normoxic conditions and treated for 24 hours with increasing concentrations of ZnCl2. (A) Untreated control; (B) 60 µM ZnCl_2_; (C) 80 µM ZnCl_2_; (D) 100 µM ZnCl_2_; and (E) 120 µM ZnCl_2_. A gradual reduction in cell confluence and density is observed with increasing zinc concentrations, particularly at 100 µM. (F) Cell viability assessed by MTS assay after 24-hour treatment with ZnCl_2_. Significant reductions were observed at 80 µM (80% ± 4%, *p < 0.05), 100 µM (60% ± 5%, ****p < 0.0001), and 120 µM (65% ± 4%, ***p < 0.001) compared to untreated controls. Data are expressed as mean ± SEM (n = 3). Statistical significance was determined using one-way ANOVA followed by Bonferroni’s post hoc test. Scale bar: 100 µm.

As an initial step to define the biological threshold of ZnCl_2_ exposure in tumor cells, we performed a cell viability assay using the MTS method. 786-0 renal carcinoma cells were treated with increasing concentrations of ZnCl_2_ (60, 80, 100, and 120 µM) for 24 hours. As shown in Figure 1A, ZnCl_2_ promoted a reduction in cell viability. Compared to the untreated control, a significant decrease was observed at 80 µM (p < 0.05), which became more pronounced at 100 µM (p < 0.0001) and 120 µM (p < 0.001).

Although multiple doses were tested in the viability screen (Figure 1A–F), 100 µM ZnCl_2_ was selected as the highest concentration that produced a significant biological response while maintaining >65% cell viability. This dose was used in all downstream experiments to ensure noncytotoxic yet functionally relevant conditions.

These findings were further supported by morphological analysis under phase-contrast microscopy (Figure 1B). Control cells (A) displayed typical morphology. At 60 µM (B), cell appearance remained unchanged. At 80 µM (C), a modest reduction in confluence and mild intercellular spacing were noted. Lower cell density became evident at 100 µM (D) and 120 µM (E), consistent with the metabolic decline detected in the MTS assay.

### Effects of Zinc on HIF-1α and VEGF-α Expression in 786-0 Cells

**Figure 2:**
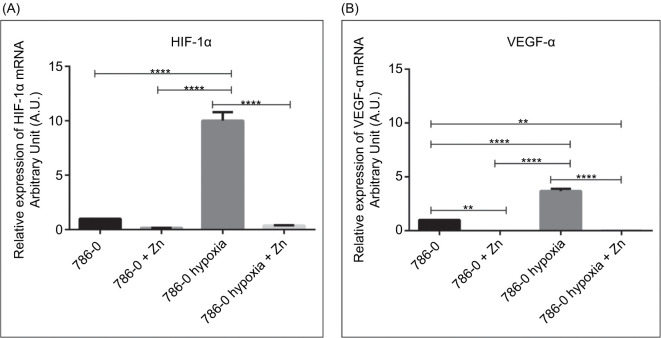
Zinc modulates HIF-1α and VEGF-α expression in 786-0 cells. (A) Relative HIF-1α and (B) VEGF-α mRNA expression levels in 786-0 renal carcinoma cells cultured under normoxic or hypoxic conditions, with or without 100 µM ZnCl_2_ treatment for 24 hours. Gene expression was analyzed by RT-qPCR and normalized to the control group. Data are presented as mean ± SEM (n = 3). Statistical significance was determined using one-way ANOVA followed by Bonferroni’s post hoc test. **p < 0.01 and ****p < 0.0001.

RT-qPCR analysis revealed that both *HIF-1*α and *VEGF-*α mRNA expression levels were modulated by hypoxia and zinc treatment in 786-0 cells (Figure 2). Under hypoxic conditions, *HIF-1*α mRNA expression showed an approximately ten-fold increase compared to normoxia (p < 0.0001). Zn supplementation reduced *HIF-1*α expression under both normoxia and hypoxia, with zinc-treated cells exhibiting levels comparable to baseline (p < 0.0001; Figure 2A).

Similarly, *VEGF-*α mRNA levels were upregulated in response to hypoxia, reaching nearly a five-fold increase compared to normoxic levels (p < 0.0001). Zinc treatment led to a decrease in *VEGF-*α expression in both oxygen conditions, with the hypoxia + Zn group showing an almost four-fold reduction compared to hypoxia alone (p < 0.0001; Figure 2B).

Western blotting was performed on 786-0 cells under normoxic and hypoxic conditions, with or without zinc supplementation, as a complementary assay to evaluate the protein levels of HIF-1α and VEGF-α.

**Figure 3:**
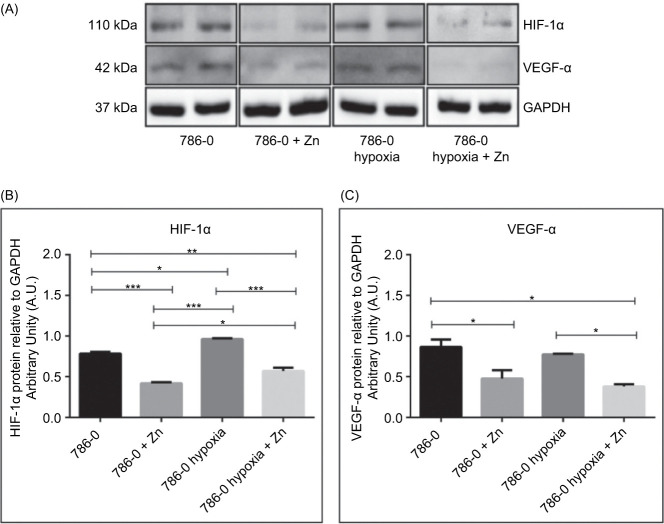
Zinc modulates HIF-1α and VEGF-α protein levels in 786-0 cells. (A) Representative Western blot showing HIF-1α (110 kDa), VEGF-α (42 kDa), and GAPDH (37 kDa) expression in 786-0 renal carcinoma cells under normoxic and hypoxic conditions, with or without 100 µM ZnCl_2_ treatment for 24 hours. GAPDH was used as the internal loading control. (B–C) Densitometric quantification of protein bands normalized to GAPDH, performed using ImageJ software and expressed in arbitrary units (A.U.). Results are expressed as mean ± SEM (n = 3). Statistical analysis was performed by one-way ANOVA followed by Bonferroni’s test. *p < 0.05; **p < 0.01; ***p < 0.001 and ****p < 0.0001.

All samples showed bands at approximately 110 kDa for HIF-1α and 42 kDa for VEGF-α, with GAPDH (37 kDa) used as the internal loading control (Figure 3A). Densitometric quantification of the immunoblot band data was consistent with the RT-qPCR findings, although the magnitude of change was greater at the mRNA level. HIF-1α protein expression increased by approximately 12% in the hypoxia group compared to normoxia (p < 0.01; Figure 3B). Zinc treatment under hypoxic conditions reduced HIF-1α levels by 40% compared to hypoxia alone (p < 0.01). VEGF-α expression did not increase under hypoxia compared to normoxia. Zinc treatment under hypoxic conditions led to a reduction of approximately 25% in VEGF-α levels relative to the hypoxia group (p < 0.05; Figure 3C).

### Zinc Differentially Modulates VEGF-α Secretion Over Time Under Normoxia and Hypoxia

To investigate the temporal regulation of VEGF-α secretion modulated by Zn under different oxygen tensions, ELISA assays were conducted using the culture supernatants of 786-0 cells treated with 100 µM ZnCl_2_ for 24, 48, or 72 hours under normoxic and hypoxic conditions.

**Figure 4:**
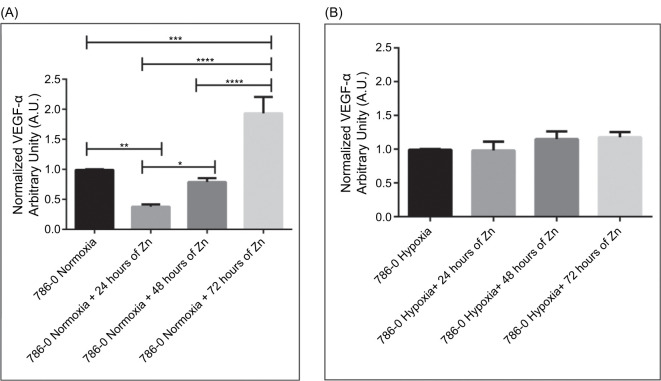
Temporal modulation of VEGF-α secretion by Zn differs between normoxia and hypoxia. (A) VEGF-α levels in 786-0 cell culture supernatants under normoxic conditions following treatment with 100 µM ZnCl_2_ for 24, 48, or 72 hours. ELISA quantification revealed a significant decrease at 24 hours (**P < 0.01), followed by a progressive increase at 48 (*P < 0.05) and 72 hours (****P < 0.0001), suggesting a biphasic response. (B) Under hypoxic conditions, VEGF-α secretion remained stable across all time points, with no significant changes upon zinc treatment. Values are normalized to respective untreated controls and expressed in arbitrary units (A.U.). Data represent mean ± SEM (n = 3). Statistical significance was determined by one-way ANOVA followed by Bonferroni’s test. *p < 0.05; **p < 0.01; ***p < 0.001; and ****p < 0.0001.

Under normoxia (Figure 4A), VEGF-α secretion was altered by zinc in a time-dependent manner. A two-fold reduction was observed at 24 hours posttreatment compared to untreated controls (p < 0.01), suggesting an early inhibitory effect. At 48 hours, an increase was observed relative to the 24-hour group (p < 0.05), and levels continued to rise, reaching a two-fold elevation over untreated normoxic cells at 72 hours (p < 0.0001). This pattern may indicate a compensatory angiogenic response following sustained zinc exposure.

In contrast, under hypoxic conditions (Figure 4B), VEGF-α secretion remained stable over time, with no statistically significant differences between control and zinc-treated groups. Although an upward trend was noted, Zn treatment appeared to mitigate the typical VEGF-α surge observed in hypoxia. These results suggest that zinc may differentially regulate VEGF-α secretion depending on oxygen availability, with more dynamic responses under normoxia that may be independent of canonical HIF signaling pathways.

### Immunofluorescence Reveals Distinct Zinc-Mediated Patterns of HIF-1α and VEGF-α Localization

**Figure 5:**
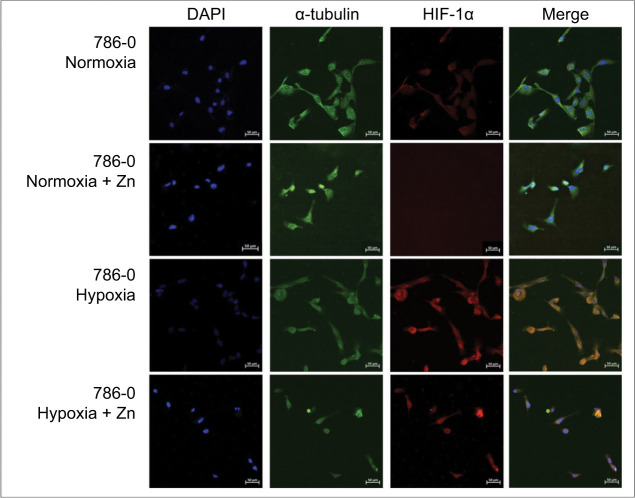
Immunofluorescence analysis of HIF-1α distribution in 786-0 cells treated with zinc under different oxygen conditions. Representative immunofluorescence images of 786-0 renal carcinoma cells cultured under normoxic or hypoxic conditions, with or without Zn treatment (ZnCl_2_, 100 µM, 24 h). Cells were stained for HIF-1α (red), α-tubulin (green), and nuclei (DAPI, blue). Under hypoxia, HIF-1α exhibited strong nuclear accumulation, which was markedly reduced by zinc treatment. Original magnification: 20×. Scale bar: 50 µm.

Immunofluorescence data revealed distinct localization patterns of HIF-1α in 786-0 cells under normoxic and hypoxic conditions with or without zinc treatment (Figure 5). Under normoxia, HIF-1α showed mild nuclear and cytoplasmic staining. Upon Zn exposure, fluorescence intensity was markedly reduced, especially in the nucleus, suggesting HIF-1α destabilization.

In hypoxia, robust nuclear accumulation of HIF-1α was observed, whereas zinc treatment suppressed nuclear signal intensity, indicating that Zn may interfere with HIF-1α stabilization even under oxygen-deprived conditions (Figure 5, merge panel).

**Figure 6:**
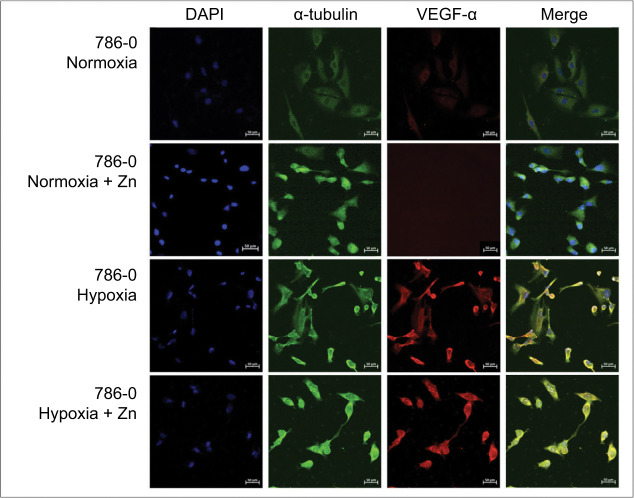
Immunofluorescence analysis of VEGF-α distribution in 786-0 Cells treated with Zn under different oxygen conditions. Representative immunofluorescence images of VEGF-α (red), α-tubulin (green), and nuclei (DAPI, blue) in 786-0 cells cultured under normoxic or hypoxic conditions, with or without zinc treatment (ZnCl_2_, 100 µM, 24 hours). Under normoxia, VEGF-α exhibited diffuse cytoplasmic staining that was markedly reduced following zinc supplementation. In contrast, under hypoxia, VEGF-α staining remained prominent in spite of Zn exposure. Original magnification: 20×. Scale bar: 50 µm.

Immunofluorescence analysis also demonstrated a zinc-mediated modulation of VEGF-α expression in 786-0 cells under both normoxic and hypoxic conditions (Figure 6). In normoxia, VEGF-α showed diffuse cytoplasmic localization, which was almost abolished after 24 hours of Zn exposure.

In contrast, under hypoxia, VEGF-α expression remained detectable in spite of Zn treatment. However, immunofluorescence revealed a distinct redistribution pattern, with enhanced nuclear localization in zinc-treated hypoxic cells. This redistribution suggests that while total VEGF-α secretion remained stable, as confirmed by ELISA, zinc may also modulate VEGF-α trafficking or nuclear retention under hypoxic stress.

### Zinc Does Not Affect Endothelial Cell Viability or Morphology

**Figure 7:**
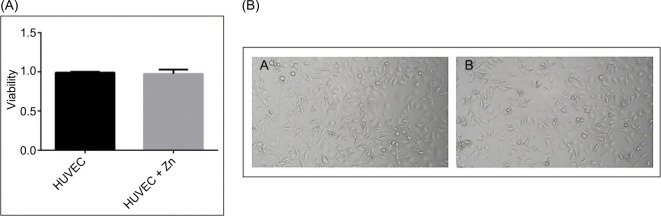
Zinc chloride does not alter HUVEC morphology or viability. (A) Cell viability of HUVECs after treatment with 100 µM ZnCl_2_ for 24 hours in RPMI 1640 medium, assessed by MTS assay. No significant differences were observed between zinc-treated and untreated groups. Data are expressed as mean ± SEM (n = 3). (B) Representative phase-contrast microscopy images of HUVECs after 24 hours of incubation with control medium (7B-A) or medium supplemented with 100 µM ZnCl_2_ (7B-B). No detectable alterations in cell morphology or density were observed. Scale bar: 100 µm.

To determine whether ZnCl_2_ directly affects the viability or morphology of endothelial cells, independent of any indirect factors, HUVECs were incubated with 100 µM ZnCl_2_ in RPMI 1640 medium for 24 hours. Phase-contrast microscopy revealed no alterations in cell structure (Figure 7B). Consistently, MTS assays showed no significant difference in cell viability between zinc-treated and control groups (Figure 7A), indicating that Zn is not directly cytotoxic to HUVECs under the tested conditions.

### Conditioned Media From Zinc-Treated Tumor Cells Suppress HUVEC Proliferation

**Figure 8:**
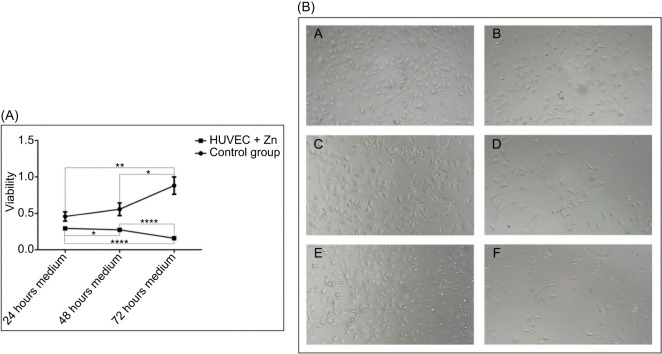
Conditioned media from zinc-treated tumor cells progressively impair HUVEC viability. (A) Viability of HUVECs exposed for 24 hours to conditioned media from 786-0 cells treated with 100 µM ZnCl_2_ for 24, 48, and 72 hours, assessed using the MTS assay. Endothelial viability progressively decreased with increasing tumor cell conditioning time, with significant reductions compared to controls. Mean viability was approximately 85% ± 4% after 24 hours, 40% ± 5% after 48 hours, and 35% ± 6% after 72 hours of exposure to zinc-conditioned media (****p < 0.0001 vs. respective controls). Data represent mean ± SEM (n = 3). Statistical analysis was performed using one-way ANOVA followed by Bonferroni’s post hoc test (*p < 0.05; **p < 0.01 and ****p < 0.0001). (B) Representative phase-contrast microscopy images of HUVECs exposed to control or zinc-conditioned media. Panels: (7B-A) 24-hour control; (7B-B) 24-hour Zn; (7B-C) 48-hour control; (7B-D) 48-hour Zn; (7B-E) 72-hour control; (7B-F) 72-hour Zn. Reduced cell density is evident in zinc-conditioned groups, while morphology remained preserved. Scale bar: 100 µm.

To investigate the effects of zinc-treated tumor cells on endothelial viability, conditioned media were collected from 786-0 cells cultured with 100 µM ZnCl_2_ for 24, 48, and 72 hours. These media were applied to HUVECs for 24 hours. Phase-contrast microscopy revealed a progressive reduction in endothelial cell density inversely proportional to the duration of tumor cell preconditioning (Figure 8B). In spite of preserved morphology and active mitotic figures in control groups, the conditioned media from zinc-treated 786-0 cells significantly reduced HUVECs proliferation at all time points, as confirmed by MTS assays (Figure 8A). Quantitatively, endothelial viability decreased by over ~60–65% following exposure to 72-hour conditioned medium (p < 0.0001), reinforcing a possible inhibitory effect.

## Discussion

Zinc has emerged as a potential modulator of tumor progression and angiogenesis, acting through multiple levels of regulation. In clear cell renal cell carcinoma (ccRCC), where HIF-1α/VEGF-α signaling driven by VHL inactivation is a hallmark, the biological effects of zinc are not fully understood. By combining gene expression analysis, protein quantification, immunofluorescence imaging, and functional assays, our study provides evidence that zinc may suppress this angiogenic axis in 786-0 ccRCC cells under normoxic and hypoxic conditions, with effects in transcriptional and posttranslational levels.

In normoxia, zinc exposure reduced HIF-1α expression at the mRNA and protein levels, consistent with previous work by Nardinocchi et al. (11), who showed that zinc promotes HIF-1α proteasomal degradation independently of VHL. This has been attributed to enhanced activity of prolyl hydroxylase domain (PHD) enzymes, which target HIF-1α for degradation. Although this mechanism was not directly tested in our study, our findings align with it, considering the nuclear reduction of HIF-1α observed by immunofluorescence. Supporting this pathway, Wong et al. (17) demonstrated that α-ketoglutarate, a cofactor required for PHD activity, can be influenced by zinc-driven metabolic shifts. In addition, our data suggest upstream transcriptional repression of *HIF-1*α, as mRNA levels were also reduced following zinc exposure.

Under hypoxia, where PHDs are typically inhibited and HIF-1α is stabilized, we still observed partial suppression of HIF-1α by zinc. Immunofluorescence analysis showed reduced nuclear accumulation, while Western blotting and qRT-PCR confirmed moderate decreases in protein (~40%) and transcript levels (~90%), respectively. This attenuation, though less pronounced than in normoxia, may involve redox-sensitive or epigenetic mechanisms. Yang et al. (6) proposed that PHD activity can persist under hypoxia if modulated by ROS and cofactors like Fe^2+^, both of which may be affected by zinc. Zinc may also act via p53 stabilization, a mechanism shown to reduce HIF-1α expression both transcriptionally and posttranslationally (11).

VEGF-α, a canonical HIF-1α target, followed a suppression profile. In normoxia, zinc treatment reduced *VEGF-*α more at transcript levels than in protein expression, marked by a decline in VEGF-α secretion at 24 hours. This was corroborated by ELISA quantification, which showed a ~50% reduction in VEGF-α secretion at 24 hours and a rebound to nearly 200% of baseline at 72 hours (P < 0.0001), consistent with a dynamic compensatory pattern possibly mediated by mTOR reactivation or autophagy-related signaling, as reported by Yu et al. (16) and Lin et al. (18). The apparent discrepancy between transcriptional and protein level effects likely reflects temporal compensation and differential half-lives between mRNA, protein, and secreted VEGF-α. Notably, the biphasic secretion pattern (early inhibition followed by rebound) was observed only under normoxia, highlighting the complexity of VEGF regulation beyond transcriptional control.

In contrast, VEGF-α secretion under hypoxia remained relatively stable across all timepoints in spite of zinc treatment. Our qRT-PCR showed that zinc maintained *VEGF-*α expression near baseline. Immunofluorescence and ELISA data indicate that zinc maintained stable VEGF-α expression. This suggests that zinc did not strongly suppress VEGF-α output, but rather neutralized its hypoxic induction. Western blot data supported this trend, showing a modest (~25–30%) protein reduction in the hypoxia + Zn group, in alignment with the more significant (~60%) reduction in transcript levels. These findings indicate that zinc may disrupt hypoxia-induced VEGF-α expression without fully repressing baseline levels, possibly through chromatin remodeling or inhibition of HIF-1α coactivators.

Immunofluorescence further revealed distinct VEGF-α localization patterns. In normoxia, VEGF-α was cytoplasmic and almost abolished after zinc exposure. In hypoxia, VEGF-α staining persisted with strong cytoplasmic distribution. Interestingly, zinc-treated hypoxic cells exhibited enhanced nuclear colocalization of VEGF-α. Although fluorescence intensity was not quantified, the observed nuclear redistribution of VEGF-α in zinc-treated hypoxic cells was consistently noted across biological replicates, supporting a reproducible pattern-level shift in localization. Our rationale was to emphasize localization patterns rather than absolute signal intensity, since nuclear versus cytoplasmic redistribution of HIF-1α and VEGF-α provides biological insights. To avoid drawing unwarranted conclusions, we confined our analysis to reproducible spatial patterns and noted that complementary semi-quantitative approaches, such as ImageJ, could strengthen future studies. While the functional implications of this redistribution remain speculative, it suggests that zinc may alter VEGF-α trafficking or nuclear retention under stress, potentially affecting its signaling dynamics.

Importantly, functional data from HUVECs support a role for zinc in impairing angiogenic signaling. While direct exposure to 100 µM ZnCl_2_ for 24 hours had no effect on HUVEC viability, conditioned media from zinc-treated 786-0 cells significantly impaired endothelial proliferation, with ~60–65% reduction after 72-hour exposure. These findings may indicate that zinc-modulated tumor secretomes exert paracrine inhibitory effects on endothelial cells. However, as VEGF-α was not neutralized or supplemented in these assays, we cannot rule out the involvement of other zinc-regulated soluble factors in mediating this effect.

An additional mechanistic layer may involve intracellular zinc trafficking. Our previous work (19) and ongoing analyses suggest that zinc treatment upregulates ZIP11 and downregulates ZnT1 in 786-0 cells, potentially increasing intracellular and nuclear zinc pools. This shift has been associated with epigenetic modulation, including enhanced p53 stabilization and suppression of angiogenic genes (11,12). Although not the focus of the current study, such transporter shifts may explain the transcriptional silencing observed for both HIF-1α and VEGF-α.

This study has limitations. The anti-angiogenic effects of zinc were assessed by a single concentration (100 µM), though this dose was selected based on viability curves identifying it as effective yet noncytotoxic. In addition, VEGF-α was not blocked or supplemented to confirm causality in HUVEC assays. Finally, the direct effects of prolonged zinc exposure (>24 h) on HUVECs were not evaluated and may be relevant for clinical translation.

## Conclusions

In conclusion, zinc may exert multilevel inhibitory effects on the HIF-1α/VEGF-α axis in ccRCC cells under both normoxia and hypoxia. These effects may involve transcriptional repression, protein destabilization, and functional disruption of paracrine angiogenic signaling. While further mechanistic validation is warranted, our findings suggest that zinc may represent a promising adjuvant candidate in anti-angiogenic therapy, particularly in tumors characterized by hypoxic adaptation.
